# Comparing the Activity of Peripheral Blood Mononuclear Cells Frozen Under Electromagnetic Field Freezing and Standard Slow‐Freezing

**DOI:** 10.1155/bmri/9884345

**Published:** 2025-11-20

**Authors:** Takehiro Matsubara, Mina Takagi, Takahiro Uwabo, Junichi Soh, Shinichi Toyooka, Mizuki Morita

**Affiliations:** ^1^ Okayama University Hospital Biobank, Okayama, Japan; ^2^ Faculty of Health Sciences, Okayama University Medical School, Okayama, Japan, okayama-u.ac.jp; ^3^ Department of Biorepository Research and Networking, Okayama University Graduate School of Medicine, Dentistry and Pharmaceutical Sciences, Okayama, Japan, okayama-u.ac.jp; ^4^ Department of Thoracic Surgery, Osaka Metropolitan University Graduate School of Medicine, Osaka, Japan, osaka-cu.ac.jp; ^5^ Department of General Thoracic Surgery and Breast and Endocrinological Surgery, Okayama University Graduate School of Medicine, Dentistry and Pharmaceutical Sciences, Okayama, Japan, okayama-u.ac.jp; ^6^ Department of Biomedical Informatics, Okayama University Graduate School of Interdisciplinary Science and Engineering in Health Systems, Okayama, Japan, okayama-u.ac.jp

## Abstract

Peripheral blood mononuclear cells (PBMCs) are cells obtained from the blood that are used not only in clinical tests but also in various research applications. The slow‐freezing (SLF) method, currently the standard for PBMC cryopreservation, involves extended storage at −80°C before transfer to liquid nitrogen. Delays in this transfer, such as overnight or weekend holds, risk a gradual decline in cell viability. Additionally, variability in freezing duration can lead to inconsistent cell quality, emphasizing the need for an alternative freezing method that allows for more timely transfer to liquid nitrogen. This study is aimed at clarifying whether the method of using a freezer with an applied electromagnetic field (EMF) is superior to the currently used standard SLF method for PBMC cryopreservation. A comparison of the number of viable cells, cell viability, and cell activity showed that the EMF method was equivalent to the SLF method. However, the shortest time required for freezing was significantly shorter with the EMF method than the SLF method (0.25 vs. 3 h), allowing for earlier transfer of PBMC to liquid nitrogen. This demonstrates that the EMF method offers an advantage in operational efficiency, particularly for facilities that routinely process and store PBMCs, such as biobanks and other storage‐focused departments.

## 1. Introduction

Human peripheral blood mononuclear cells (PBMCs) are a group of cells isolated from the blood that have various applications, such as immunological research [1–3], DNA/RNA extraction [4, 5], generation of iPS cells [6], epigenetics [7], mRNA/miRNA signatures [8], and collection of circulating tumor cells (CTCs) [9]. When prepared PBMCs are used as viable cells for culture or other purposes, it is desirable to use them immediately after preparation; however, if this is not possible, they are frozen and stored. In this case, the sample quality may decline during freezing, storage, and thawing processes [10, 11]. To minimize this, liquid nitrogen (approximately −150°C) is chosen as the cryopreservation temperature range for PBMC. During thawing, the buffer solution should be changed quickly to minimize the adverse effects of cryoprotectants such as dimethyl sulfoxide (DMSO). However, the optimal freezing method differs depending on the cell type [12], whereas the slow‐freezing (SLF) method was chosen for PBMC.

In the SLF method of cells, the temperature is lowered at a rate of 1°C/min, and programmable freezers and cell freezing containers are used for this gradual freezing, with the latter in particular being widely used because of its cost and flexibility advantages. In the SLF method using cell freezing containers, tubes containing PBMCs are placed in a cell freezing container and frozen in a deep freezer at −80°C. After the PBMCs drop to −80°C, they are transferred to liquid nitrogen (−150°C). If PBMC are kept at −80°C for too long, the quality of viable cells will gradually decline during that time. Therefore, it is best to transfer the cells to liquid nitrogen as soon as possible after the temperature drops to −80°C. However, if blood samples are collected and processed in the evening and then frozen at −80°C, transferring the PBMC to liquid nitrogen will occur late in the day. In this case, the staff may either stay until the late hours or transfer the PBMC to liquid nitrogen the following day. In the latter case, the PBMC would be kept at −80°C for 16 h, for example. If the blood samples are collected on a Friday or before a holiday weekend, they will be kept at −80°C for several days. This is also undesirable because the quality will not be uniform due to the variation in the time at which the blood is kept at −80°C, depending on the time of day and the day when the blood is collected. Therefore, a freezing method that allows transfer to liquid nitrogen in a shorter time is required.

In recent years, it has been reported that freezing in the presence of a static magnetic field and/or static electric field during the freezing and storage of foodstuffs can maintain high freshness, and there have been reports of the application of such freezing methods to the preservation of living cells [13, 14]. Freezing in the presence of magnetic and electric fields can shorten the freezing time or eliminate the use of cryoprotectants, thereby improving the quality and efficiency of cell storage. As mentioned above, there have already been reports of attempts to store cells; however, because the optimal freezing method depends on the cell type [12], it is not possible to determine whether this freezing method is effective in PBMC based on the results of other cell types.

This study is aimed at clarifying whether freezing PBMC in an electromagnetic field (EMF) freezer is superior to the standard SLF procedure using cell freezing containers in terms of the number of viable cells, viability, cell activity, and time required for freezing.

## 2. Materials and Methods

### 2.1. Sample Collection

Blood was drawn from healthy volunteers (*n* = 5) into EDTA‐containing tubes (EDTA‐2K, 6 mL; Becton Dickinson, Franklin Lakes, New Jersey, United States) at the Okayama University Hospital Biobank (Okadai Biobank). This study was performed per the Declaration of Helsinki and was approved by the Ethics Committee of Okayama University (approval number K1708‐042). All the individuals provided written informed consent to participate in the study. The blood samples were processed immediately after collection.

### 2.2. Determination of the Minimum Freezing Time in the EMF Freezer

To determine the minimum freezing time of EMF freezing, the temperature in a 0.5‐mL sample storage tube (Thermo Fisher Scientific, Waltham, Massachusetts, United States) filled with cryopreservation medium Bambanker hRM (Nippon Genetics, Tokyo, Japan) was monitored with a calibrated temperature probe immediately after loading into the EMF chamber (−30°C). The elapsed time until the sample core reached −30°C was recorded and used to define the minimum freezing time. Temperatures were measured at four chamber positions (left and right sides of the top shelf and left and right sides of the bottom shelf).

### 2.3. PBMC Preparation and Cryopreservation

All the PBMC preparation steps were performed at room temperature. The contents of the blood collection tubes from all five individuals were combined into a single 50‐mL conical tube, mixed, and then aliquoted into 6 mL each into five tubes. Each aliquot was layered onto 3 mL Ficoll‐Paque PLUS (Cytiva, Tokyo, Japan) in a 15‐mL tube and subsequently centrifuged at 400 × *g* for 30 min at room temperature with fast acceleration and low brake. The ring‐shaped interphase PBMCs were collected with a micropipette into a new 15‐mL tube, and 5 mL of D‐PBS(‐) (Takara Bio, Shiga, Japan) was added. The tube was centrifuged at 400 × *g* for 10 min at room temperature (20°C–25°C) with rapid acceleration and braking. After centrifugation, the supernatant was discarded. The cells were resuspended in 5 mL of D‐PBS(‐) and centrifuged again at 400 × *g* for 10 min at room temperature with rapid acceleration and braking. The supernatant was discarded. The cells were resuspended in Bambanker hRM, and the total cell number, number of living cells, and percentage of total living cells were determined using an automatic cell counter, TC20 (Bio‐Rad, Hercules, California, United States), with trypan blue staining (1:1), per the manufacturer′s instructions. Bambanker hRM was added at a concentration of 4 × 10^6^ cells/mL. The PBMC suspension was dispensed into 0.5‐mL tubes at 520 *μ*L each. PBMC suspensions were frozen using a freezer that applied an EMF (Proton Freezer PF‐15A, Ryoho Freeze Systems, Nara, Japan) (EMF method) or a SLF procedure with cell freezing containers (BICELL, Nihon Freezer, Tokyo, Japan) (SLF method). In the SLF method, the tubes were frozen for 3 h, which is the minimum freezing time recommended by the manufacturer, or 168 h at −80°C. In the EMF method, the tubes were frozen directly for 0.25 h, which was the minimum freezing time determined by experiments with water, or 168 h. After each freezing period, all tubes were transferred into a cryotank (LS6000, Worthington Industries) and stored in the liquid nitrogen vapor phase for 30 days.

### 2.4. PBMC Thawing

PBMC‐containing tubes were placed in a prewarmed water bath (37°C) until only 2–3 mm cubes remained. They were then removed from the water bath, and the PBMC suspensions were completely dissolved at room temperature. The PBMC suspensions were mixed by gentle pipetting, and the contents of the tubes were transferred into a 15‐mL conical tube. A total of 9 mL of cell culture media (FBS [×10] and penicillin–streptomycin [x100]–supplemented RPMI media) was added slowly. PBMC suspensions were centrifuged at 400 × *g* for 5 min at room temperature, and the pellet was resuspended in the same medium. These washing steps were repeated twice for each sample. Subsequently, the PBMCs were counted using TC20 with trypan blue staining (1:1) and resuspended in a volume adjusted for the assay.

### 2.5. PBMC Proliferation Assay

The 96‐well cell culture plates were coated with a poly‐L‐ornithine solution (0.01%, 50 *μ*L/well) (Sigma‐Aldrich, St. Louis, Missouri, United States) and incubated at ambient temperature for 1 h. The solutions were aspirated from the wells, and the plates were allowed to dry at ambient temperature for 30–60 min. After the plates were dried, frozen PBMCs were thawed and counted as described above. PBMCs were resuspended in a cell culture medium at a concentration of 4 × 10^5^ cells/mL. After the 96‐well cell culture plates had dried, immune cell activation treatments were prepared by diluting both anti‐CD3 Ab (Invitrogen, Waltham, Massachusetts, United States) and anti‐CD28 Ab (Invitrogen) to a concentration of 2 *μ*g/mL (2× final assay concentration of 1 *μ*g/mL) and adding 100 *μ*L to each well before cell addition. Additionally, a cell culture medium without activators was added to the wells as a control. Then, 100 *μ*L of PBMC suspensions was seeded into every well of the cell plate (40,000 cells/well). PBMCs were allowed to settle at ambient temperature on a level surface for at least 30–60 min to ensure even distribution and adherence of cells. The cell plates were carefully placed into an IncuCyte ZOOM instrument (Sartorius), and repeated scanning was performed for 7 days.

### 2.6. Cytokine Measurement in PBMC Supernatant

Cytokine levels were determined in the supernatants collected from the PBMC proliferation assays (anti‐CD3 Ab–stimulated and anti‐CD28 Ab–stimulated cells) after 7 days. All cytokine measurements were carried out with the Bio‐Plex Pro Human Cytokine Group I Panel 8‐Plex (Bio‐Rad) per the manufacturer′s instructions. Briefly, the supernatant samples were incubated with captured antibody‐coupled magnetic beads. After three washes with a Bio‐Rad Wash Station II, the samples were incubated with a biotinylated detection antibody. Each captured cytokine was detected by adding streptavidin–phycoerythrin. Averages and standard deviation (SD) were calculated from the data using Excel.

### 2.7. Statistical Analysis

For the proliferation assays, each condition was performed in triplicate (*n* = 3 independent replicates). For the cytokine assays, each condition was performed in duplicate (*n* = 2). Data in graphs are presented as means, and error bars denote SDs.

## 3. Results

### 3.1. Minimum Freezing Time in the EMF Freezer

**Figure 1:**
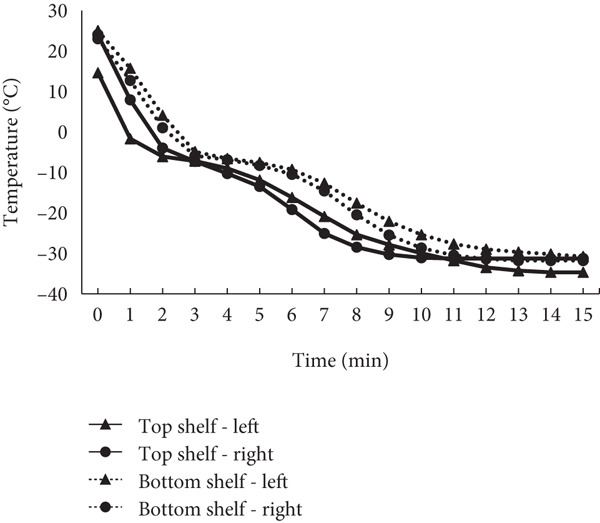
Temperature profiles of sample tubes after loading into the EMF freezer (−30°C). The minimum freezing time for this study was set to 15 min (0.25 h) based on the time required for the sample, measured with a temperature probe in a 0.5‐mL storage tube filled with cryopreservation medium, to reach −30°C.

As shown in Figure 1, the sample temperature reached −30°C within 15 min (0.25 h). No appreciable positional difference in time‐to‐set‐point was observed across the four chamber positions. Accordingly, 15 min (0.25 h) was adopted as the minimum freezing time prior to transfer to liquid nitrogen.

### 3.2. Counts and Percentage of Live Cells

The counts and percentages of live cells were compared between the EMF and SLF methods to investigate their effect on cell viability (Figure 2). In the case of the minimum recommended or predetermined freezing time (EMF: 0.25 h; SLF: 3 h), the SLF method showed a slight decrease in both the count of live cells (Figure 2a, left side; EMF: 4.16 × 10^6^ cell/mL; SLF: 3.91 × 10^6^ cell/mL) and the percentage of live cells (Figure 2b, left side; EMF: 79.0%; SLF: 74.0%) compared to the EMF, but these differences were not significant.

Figure 2Assessment of viable PBMCs after freezing with the EMF and the SLF methods evaluated with (a) the cell counts and (b) the cell proportion. For both evaluation indices, a comparison of the results obtained when frozen at the shortest freezing time (EMF: 0.25 h; SLF: 3 h) for each of the EMF and SLF methods and when frozen over a longer period (168 h) is shown.

**Figure:**
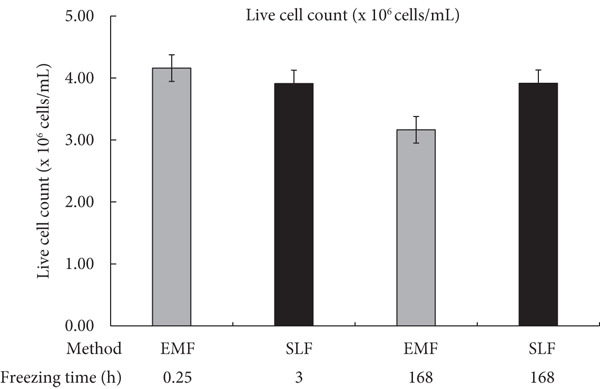
(a)

**Figure:**
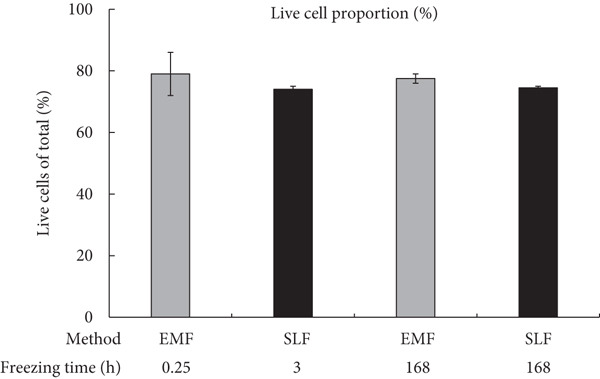
(b)

After a long freezing time, no significant change in the percentage of live cells was detected (Figure 2b, right side; EMF: 77.5%; SLF: 74.5%). However, the live cell counts in the long freezing time using the EMF method were lower than those at the minimum recommended freezing time, whereas the SLF method showed the same count for both freezing durations (Figure 2a, right side; EMF: 3.17 × 10^6^ cell/mL; SLF: 3.92 × 10^6^ cell/mL).

### 3.3. Proliferation Assay

A proliferation assay was conducted under activation conditions using anti‐CD3 and anti‐CD28 antibodies to demonstrate their effects on cell activity (Figure 3). In the case of the minimum recommended freezing time, both methods demonstrated similar cell growth levels (Figure 3a,b; EMF: 8.2%; SLF: 6.5%), although the SLF method exhibited a larger deviation compared to the EMF method (EMF: ±0.76%; SLF: ±2.67%). However, a long freezing time resulted in a severe negative effect on the growth ability (Figure 3c,d). Using the SLF method, the cells exhibited growth, but this condition also demonstrated a wide deviation, similar to that of the minimum recommended freezing time.

Figure 3Proliferation assay of PBMCs after freezing with (a) EMF 0.25 h, (b) SLF 3 h, (c) EMF 168 h, and (d) SLF 168 h under the stimulation of anti‐CD3 and anti‐CD28 Abs, which was conducted to evaluate the effect of freezing method (EMF and SLF) and duration (shortest and longer times) to cell viability.

**Figure:**
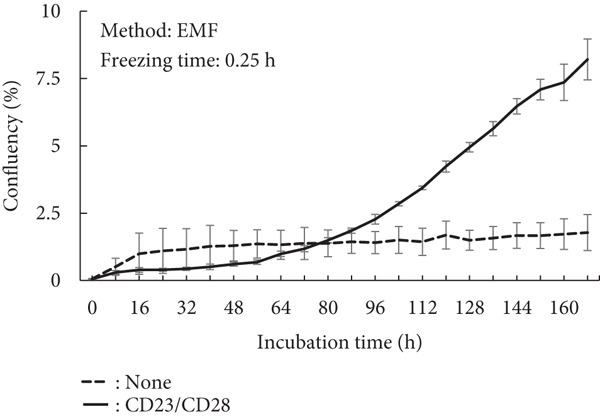
(a)

**Figure:**
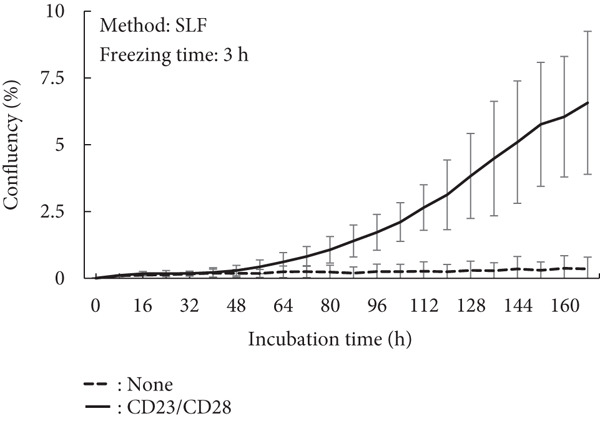
(b)

**Figure:**
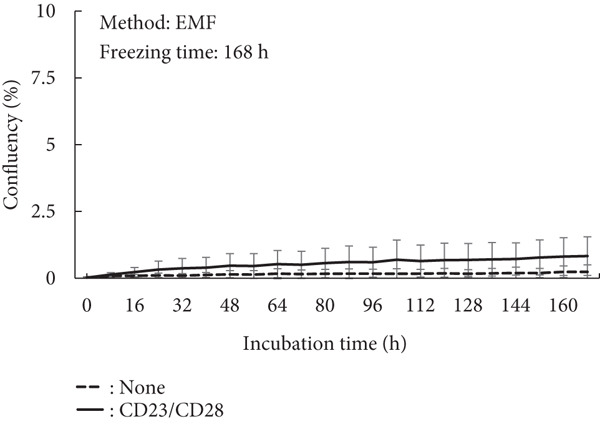
(c)

**Figure:**
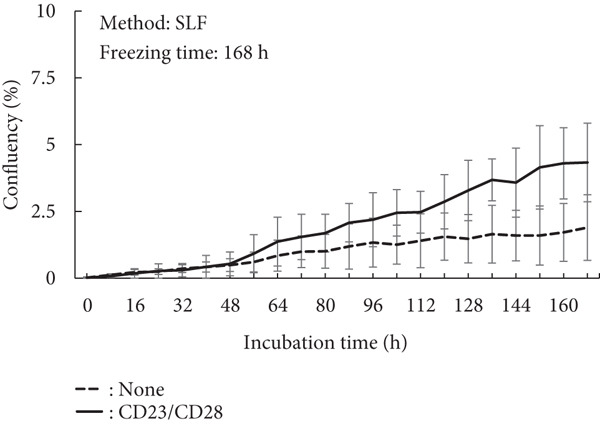
(d)

### 3.4. Cytokine Production

**Figure 4:**
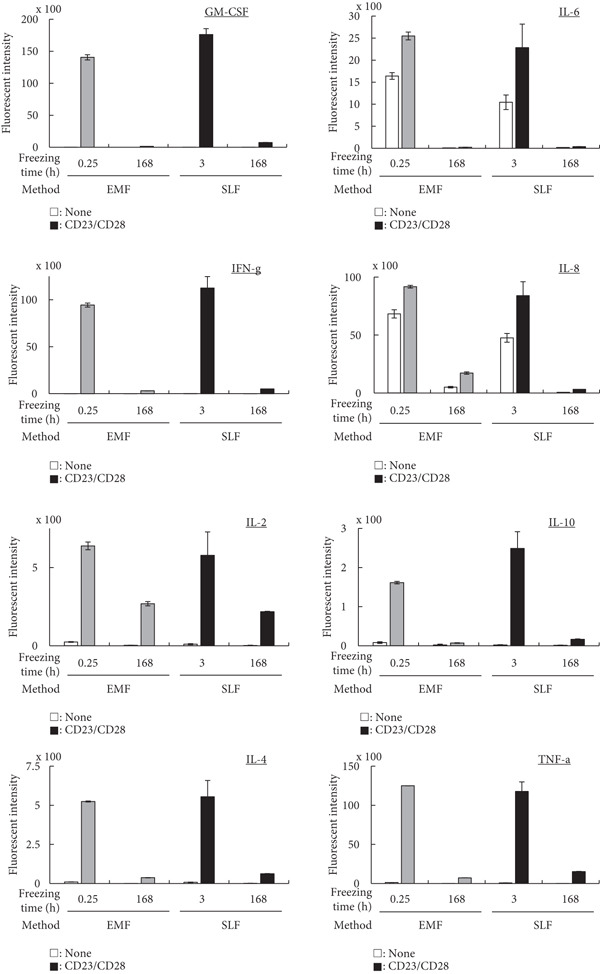
Measurement of cytokine production of PBMCs after freezing with the EMF and SLF methods under the stimulation of anti‐CD3 and anti‐CD28 Abs, which was conducted to evaluate the effect of freezing method (EMF and SLF) and duration (shortest and longer times) to cell activity.

A cytokine production assay was performed to examine its effect on one of the important functions of PBMCs (Figure 4). At the minimum recommended freezing time, both methods demonstrated favorable cytokine production under the activation of anti‐CD3 and anti‐CD28 Abs, with no noticeable difference between the conditions. For example, the IL‐4 fluorescence intensity was 10 without CD23/CD28 stimulation and 520 with stimulation for the EMF method, whereas it was 10 and 550 for the SLF method. However, with a long freezing time, cytokine production was remarkably reduced for many cytokines in both freezing methods. For the EMF, IL‐4 fluorescence intensity was 0 without stimulation and 40 with stimulation. For the SLF, the values were 0 and 60.

## 4. Discussion

The minimum freezing was 0.25 h for the EMF method and 3 h for the SLF method. Therefore, the EMF method can transfer specimens to liquid nitrogen 2 h and 45 min earlier than the SLF method. This means that, for example, if blood samples are collected in the evening, the EMF method allows the technical staff to return home much earlier. This might not be a problem for certain studies that require a few PBMCs; however, for departments that routinely store PBMCs (e.g., biobanks), this has an impact.

When PBMCs were frozen using the EMF and SLF methods, the number and percentage of viable cells were similar when frozen for the shortest time (0.25 and 3 h, respectively) (Figure 2a,b, left side in each graph), and similar trends in cell proliferation and cytokine production under antibody stimulation were also observed (Figures 3a,b and 4, left side in each condition). In contrast, when PBMCs were kept in the EMF freezer or frozen container for 7 day (168 h), the number and percentage of viable cells remained almost unchanged compared to the shortest time in both cases (Figure 2, right side in each graph), but the cellular activity quality was significantly reduced (Figures 3c,d and 4, right side in each condition). Therefore, when storing PBMCs as viable cells, it is necessary to transfer them to liquid nitrogen as soon as possible after freezing at the respective minimum times for both freezing methods.

One of the considerations during the cell freezing process is preventing cell damage by ice nucleation and ice crystal growth inside and outside the cell [11]. In the SLF method, intracellular water moves out of the cell before freezing, thereby inhibiting the formation of ice crystals [15, 16]. According to the manufacturer, the EMF freezer used in this study generates a static electric field and a static magnetic field (uniform magnetic flux density) in the chamber, and cold air is delivered to accelerate the temperature decrease of the object to be frozen. It is known that the application of an electric or magnetic field during freezing inhibits cell damage [17–19], but the detailed mechanism is unclear, and the results reported in the literature are contradictory [14]. The magnetic flux density inside this EMF freezer was not disclosed by the manufacturer but was measured to be 2.03 mT (SD 0.28 mT). This is a fairly low flux density, as discussed previously [19, 20], and the effect of this magnetic field is unknown.

As we have discussed, our results indicate that the EMF method used in this study has the same freezing performance in PBMCs as the standard SLF method. As described above, this effect may be achieved through three factors: a static electric field, a static magnetic field, and cold air. However, from our results, it is not possible to determine what contributed to this effect and to what extent. If we could stop the electric field, magnetic field, and cold air individually, we would be able to clarify the effect of each; however, this is not possible because the equipment we use is a ready‐made product. If the involvement and contribution of each of these factors can be clarified, it can be expected to lead to optimization of freezing methods. Further research is required to elucidate the underlying mechanisms.

## 5. Conclusions

In summary, freezing PBMCs by the EMF method used in this study resulted in viable cells, viable cell rate, and cell activity comparable to the standard SLF method. Nevertheless, the time required to transfer the PBMCs to liquid nitrogen was significantly shorter with the EMF method than with the SLF method. The EMF method showed an equivalent number of viable cells, viability, and cell activity as the SLF method in a much shorter time. Based on these results, we concluded that the EMF method can improve the operational efficiency of biobanks and other institutions that freeze and store PBMCs as part of their services. Thus, the EMF method can benefit biobanks in the routine processing and storage of PBMCs. However, further research is needed to determine whether magnetic and electric fields are responsible for the cytoprotective mechanism of the EMF freezer used in this study.

## Ethics Statement

This study was performed per the Declaration of Helsinki and was approved by the Ethics Committee of Okayama University (approval number K1708‐042).

## Consent

All the individuals provided written informed consent to participate in the study.

## Conflicts of Interest

We were lent an EMF freezer free of charge by Hayashiroku.

## Author Contributions

Takehiro Matsubara: experiment, data curation, statistical analysis, visualization, and writing—original draft. Mina Takagi: experiment, data curation, and visualization. Takahiro Uwabo: experiment, data curation, and visualization. Junichi Soh: conceptualization, methodology, and project administration. Shinichi Toyooka: supervision. Mizuki Morita: conceptualization, supervision, and writing—review and editing.

## Funding

This study was funded by the Japan Agency for Medical Research and Development, 10.13039/100009619, JP24kk0305023.

## Data Availability

The data that support the findings of this study are available from the corresponding author upon reasonable request.
